# Targeting Epigenetic Processes in Photodynamic Therapy-Induced Anticancer Immunity

**DOI:** 10.3389/fonc.2015.00176

**Published:** 2015-07-30

**Authors:** Malgorzata Wachowska, Angelika Muchowicz, Jakub Golab

**Affiliations:** ^1^Department of Immunology, Medical University of Warsaw, Warsaw, Poland

**Keywords:** photodynamic therapy, cancer, immunotherapy, epigenetic mechanisms, histone deacetylase, methyltransferase

## Abstract

Photodynamic therapy (PDT) of cancer is an approved therapeutic procedure that generates oxidative stress leading to cell death of tumor and stromal cells. Cell death resulting from oxidative damage to intracellular components leads to the release of damage-associated molecular patterns (DAMPs) that trigger robust inflammatory response and creates local conditions for effective sampling of tumor-associated antigens (TAA) by antigen-presenting cells. The latter can trigger development of TAA-specific adaptive immune response. However, due to a number of mechanisms, including epigenetic regulation of TAA expression, tumor cells evade immune recognition. Therefore, numerous approaches are being developed to combine PDT with immunotherapies to allow development of systemic immunity. In this review, we describe immunoregulatory mechanisms of epigenetic treatments that were shown to restore the expression of epigenetically silenced or down-regulated major histocompatibility complex molecules as well as TAA. We also discuss the results of our recent studies showing that epigenetic treatments based on administration of methyltransferase inhibitors in combination with PDT can release effective mechanisms leading to development of antitumor immunity and potentiated antitumor effects.

## Introduction

Photodynamic therapy (PDT) is a light-based therapeutic approach used for the treatment of various solid tumors and non-malignant diseases. Its mechanism of action involves three non-toxic and harmless components: photosensitizer (PS), light, and oxygen. Their spatiotemporal encounter triggers photochemical reaction leading to formation of singlet oxygen and ensuing photodamage in tumor tissue (1). PS can be applied topically or administered systemically. After a period allowing for PS accumulation within the tumor, light of appropriate wavelength is precisely delivered to tumor site, usually from laser sources. Light activates PS from its ground state to the excited triplet state. Activated PS transfers its energy to molecular oxygen, leading to generation of highly reactive singlet oxygen, or reacts directly with biomolecules forming free radicals such as superoxide ion, hydroxyl radical, or hydrogen peroxide. These reactive oxygen species (ROS) mediate oxidative damage of intracellular macromolecules causing tumor cell death (2, 3).

## Photodynamic Therapy and Anticancer Immunity

Direct cytotoxic effects induced by PDT do not explain strong antitumor activity of this treatment observed in experimental animals. For example, cells from the tumors excised immediately after curative PDT are still clonogenic indicating that there must be other, indirect mechanisms triggered by PDT that contribute to the efficacy of the treatment (4). These indirect mechanisms include disruption of tumor vasculature, leading to oxygen and nutrient deprivation, and induction of robust inflammatory reaction, which can further stimulate development of antitumor immune responses (5, 6). A critical role in the therapeutic outcome of PDT is played by the immune system. Studies in immunodeficient mice or in mice inoculated with lymphocyte-depleting antibodies revealed that the presence of effector immune cells is necessary for maximum therapeutic response (7–9).

### Innate immune response in PDT

Massive PDT-induced photooxidative damage occurs mainly in the membranes and cytoplasm of tumor cells, tumor vasculature, and other stromal elements. This substantial PDT-mediated local injury threatens the host tissue integrity and homeostasis. Therefore, a host response develops as acute local inflammation in order to eliminate dead and injured cells, heal lesion, and restore tissue function as well as maintain its homeostasis (8). This response is induced almost instantaneously following PDT. Triggered by massive cell death, release of cytoplasmic components, vasoactive substances, as well as activation of the complement cascade, it leads to the secretion of leukocyte chemoattractants, cytokines, growth factors, and other immunoregulators that lead to a robust infiltration of the tissue with neutrophils, mast cells, macrophages, and NK cells (10).

A number of cytokines have been detected both within the tumor and in the plasma of mice undergoing PDT. Among a wide range of cytokines (IL-1β, IL-6, IL-10, TNF, and G-CSF) and chemokines (KC and MIP2) induced after PDT, a very important role is assigned to IL-1β and IL-6 (11). Neutralization of IL-1β reduces the cure rates of PDT-treated tumors, whereas no significant effects are observed with anti-TNF-alpha and anti-IL-6 antibodies (12). Also, recombinant cytokines such as G-CSF, GM-CSF, and TNF combined with PDT enhance antitumor response (13–15), whereas blocking anti-inflammatory cytokines such as IL-10 and transforming growth factor-β (TGF-β) improves the cure rates of PDT-treated tumors (8).

Neutrophils are the first cells invading PDT-treated tumor sites showing remarkable impact on PDT-mediated tumor damage. It was shown that neutrophils depletion in tumor bearing mice and rats attenuates the efficacy of antitumor PDT (16, 17). Monocytes/macrophages infiltrating tumor bed also seem to participate in regulating the outcomes of PDT. Inactivation of macrophages with silica particles decreases cure rates in mice, whereas treatment with macrophage-activating factor Vitamin D3-binding protein or GM-CSF potentiates antitumor effects of PDT (13, 18). Role of innate immune response in antitumor PDT is also associated with activity of NK cells. Depletion of NK cells significantly inhibits the response to PDT at suboptimal dose (19).

Innate immune cells encounter released tumor antigens (including oxidatively modified ones) together with molecules known as damage-associated molecular patterns (DAMPs) or cell death-associated molecular patterns (CDAMPs) (9). DAMPs play an analogous role to pathogen-associated molecular patterns (PAMPs), serving as warning signals. Recognition of PAMPs leads to initiation of the pathogen-induced responses, whereas DAMPs promote inflammatory responses to cell stress, injury, or death. DAMPs bind to pattern recognition receptors (PRRs) on the surface of infiltrating leukocytes and activate antigen-presenting cells (APCs) to stimulate innate and adaptive immunity. Therefore, DAMPs released from PDT-treated tumor cells are believed to be the key players in the immunogenicity of tumor cells (20, 21). The best known and frequently reported examples of PDT-induced DAMPs include heat-shock protein (HSP) family (HSP70 and HSP90), high mobility group box-1 (HMGB-1), adenosine triphosphate (ATP), and calreticulin (CRT).

### Adaptive immune response in PDT

It was demonstrated that the degree of PDT-mediated inflammation influences adaptive immune response and generation of antitumor immunity (22). The link between innate and adaptive immune response is dendritic cells (DCs), the most potent APCs, capable of migrating to secondary lymphoid tissues to prime T cells (23). PDT that triggers necrotic and apoptotic tumor cell death, accompanied by oxidative stress and induction of HSPs, is believed to shape a unique environment with tumor antigens and “danger” signals activating DC maturation (24, 25). It was shown that PDT-elicited local and systemic inflammation results in attraction of DCs to the tumor site, their activation, and maturation (24, 26, 27). DCs that have captured tumor-derived proteins and encounter DAMPs undergo activation and functional maturation, migrate to the tumor-draining lymph nodes, where they present tumor-associated antigens (TAA) in association with major histocompatibility complex (MHC) class I and II molecules to T lymphocytes. This allows selection, proliferation, and differentiation of the rare antigen-specific T lymphocytes into effector T cells (28). During effective adaptive antitumor immune response, activated T cells return to the circulation in order to home to the tumor site to carry out their effector functions (29). There are several independent studies underscoring the role of effector CD8^+^ cytotoxic T cells (CTLs) in PDT outcome. Long-term tumor control after PDT treatment is possible only in immunocompetent mice, whereas in immunodeficient SCID or nude mice the long-term effects are abrogated. However, adoptive transfer of T cells from normal mice that underwent successful PDT restores antitumor PDT efficacy in immunodeficient animals. Importantly, T-cell depletion studies revealed that the CD8^+^ T-cell population is critical for a successful PDT response whereas CD4^+^ T cell population plays only a supportive role (19).

Several reports describe the essential role of CD8^+^ T cells also in clinical PDT efficacy. Tumors lacking MHC class I on their surface are resistant to specific antitumor immune response since recognition of MHC I is necessary for CD8^+^ T cell activation (30). Moreover, PDT of multifocal angiosarcoma resulted in spontaneous regression of untreated distant tumors accompanied by increased infiltration with CD8^+^ T cells (31).

## Tumor Escape Mechanisms from Immune Surveillance

There is a strong evidence from mouse and human studies for the existence of antitumor immune response. However, tumor cells engage diverse mechanisms to modulate the immune response and to evade recognition and elimination by effector lymphocytes (32). “Cancer immunoediting” concept was proposed to describe the interactions between tumors and the immune system. According to this paradigm tumors are kept under surveillance of the immune system that either eliminates nascent tumor cells or keeps them at check in the so called equilibrium. But this protective response also shapes transformed cells in the “immunoediting” process to select for variants that develop escape mechanisms mitigating development of an effective antitumor immune response (33). A variety of mechanisms develop in tumors to avoid recognition by cells of the immune system. These mechanisms can be either inherent to tumor cells themselves or develop in stromal compartment.

### Defective antigen presentation

Presentation of TAA to lymphocytes is critically dependent on the multiple components of the antigen processing machinery (APM). It consists of four major steps: (1) peptide processing, (2) peptide transport, (3) MHC class I assembly, and (4) antigen presentation (34). A fundamental mechanism resulting from immunoediting and allowing tumor cells to evade immune surveillance is associated with down-modulation of APM. The loss of MHC class I antigens is one of the most frequent way to evade immune recognition (35, 36). Total loss of MHC I may be a result of structural changes in the β2-microglobulin gene resulting from mutations, deletion or loss of heterozygosity. Whereas decreased expression of these molecules largely depends on the regulation of transcriptional processes, involving epigenetic modulation (see below). Impaired APM can also be caused by decreased expression of proteasome subunits LMP-2 LMP-7, and LMP-10 down-regulation of proteasome activator PA28, peptide transporters TAP-1, and TAP-2 as well as chaperones tapasin and calnexin. These phenomena are observed in various types of tumors both in mice and humans, often in metastases (35, 37–39).

Another mechanism of insufficient antigen presentation involves loss or down-regulation of potentially immunogenic TAA expression. In melanoma, tumor development is frequently related to low level of TAA (32). Similarly, reduced expression of MUC-1 antigen is observed in human breast cancer cells. CD8^+^ T lymphocytes isolated from patients with low expression of MUC1 do not react to autologous tumors (40). Molecular mechanisms responsible for changes in MHC expression on tumor cells include several types of gene modifications. However, *in vitro* studies show that loss of one allele or haplotype occurs very rarely (41). Therefore, it is suggested that tumor cells engage different strategies affecting antigen presentation in order to escape from immune recognition. Recent studies emphasize the role of epigenetic changes not only in tumor development and progression but also in tumor evasion (42, 43). It seems that epigenetic modifications play a key role in regulation of MHC, APM, and TAA expression level in tumor cells.

### Tolerance, deviation, and adaptation

Although tumor cells, with rare exception of hematologic malignancies, do not express co-stimulatory molecules, they can express inhibitory molecules, such as PD-L1, PD-L2, LAG-3, TIM3, BTLA-4, or VISTA that induce deletion or anergy of tumor-reactive T cells. Some of these molecules as well as tumor-expressed FasL (CD95L/Apo1L) can also induce cell death in both T and NK cells. Another related mechanism is associated with surface expression of non-classical MHC class I molecules HLA-G and HLA-E that inhibit cytotoxic activity of effector CTLs and NK cells (44–46). Circulating MICA and MICB molecules, ligands for NKG2D receptor attenuate effector capacity of T lymphocytes and NK cells (47).

Suppressed antitumor immune response may be a result of tumor-induced changes in the function of DCs. Human and mouse tumors release cholesterol metabolites down-regulating the expression of CCR7 receptor on maturing DCs. This inhibits CCR7-dependent DC migration to lymphoid organs (48). Moreover, tumor cells produce vascular endothelial growth factor (VEGF) responsible not only for tumor angiogenesis, but also for impairment of DC maturation. Treatment with monoclonal antibodies against VEGF improves DC function *in vivo* and the efficacy of cancer immunotherapies (49). TGF-β negatively influences the activity of T lymphocytes and NK cells, inhibits maturation of DCs, and facilitates the recruitment of regulatory T cells (50). Likewise, immunosuppressive IL-10 is known to inhibit the function of APCs and generation of CTLs as well as suppress the activity and/or migration of CTLs (51).

Moreover, tumor cells can release enzymes that metabolize amino acids regulating activity of immune cells. One of such enzymes is indoleamine 2,3-dioxygenase (IDO), responsible for tryptophan catabolism. Enhanced expression of IDO in some types of tumors causes local shortage of tryptophan, leading to disturbances in proliferation of alloreactive T lymphocytes and their cell cycle arrest (52). Additionally, some tryptophan metabolites induce apoptosis in CD4^+^ T lymphocytes whereas kynurenine, a product of IDO-mediated tryptophan catabolism, leads to their differentiation into T regulatory cells (Tregs) that down-regulate immune response (53, 54).

Tumor cells that are unable to escape from immune recognition using the above mechanisms develop adaptation mechanisms to evade effector CTL-induced death. They can up-regulate expression of antiapoptotic molecules such as FLIP or BCL-X_L_ (55, 56). Otherwise, in order to avoid cell death, tumors can express inactive death receptors such as TRAIL-R1, TRAIL-R2, or FAS (57, 58).

### Immunosuppressive cells

Together with tumor-intrinsic immune escape mechanisms described above, tumors may also highjack parts of the immune system to evade immune attack. To achieve this goal, they induce or recruit immune-suppressive Tregs as well as myeloid-derived suppressor cells (MDSC), which under normal conditions serve as safeguards against overwhelming inflammation. In this way, tumors turn the immune system against itself, and exercise a powerful arsenal of mechanisms unavailable to tumor cells themselves to mitigate anti-tumor immune activity. Tregs inhibit activation and expansion of antigen-specific CD8^+^ T lymphocytes, through high expression of immune-inhibitory receptors cytotoxic T-lymphocyte-associated antigen-4 (CTLA-4) and PD-L1, secretion of immunosuppressive cytokines such as IL-10 and TGF-β, and by consuming IL-2 (59). There are also other regulatory populations of lymphocytes that can be found among subsets of B cells and NKT cells inhibiting antitumor effector cell responses (60). MDSCs are heterogeneous population of cells originating from bone marrow including progenitor and immature myeloid cells of granulocytic or monocytic lineages (61). MDSCs engage several diverse strategies to suppress tumor growth by inhibiting tumor cell cytotoxicity mediated by NK cells and by blocking the activation of tumor-reactive CD4^+^ and CD8^+^ T cells (62, 63). These mechanisms include production of immunosuppressive cytokines such as TGF-β and IL-10, production of reactive nitrogen and oxygen species, interference with T cell homing, and contribution to tumor angiogenesis (61, 63, 64). Moreover, MDSCs prevent antigen/MHC peptide recognition by nitrosylation of T cell receptor (TCR) and deplete amino acids such as tryptophan (IDO) or arginine (arginase-1) that are required for activation and proliferation of T cells (65). Additionally, MDSCs induce accumulation of Tregs, which in turn down-regulate cell-mediated immunity and promote a Th2 type response that favors tumor progression (66).

## The Role of Epigenetic Changes in Immune Escape

**Figure 1:**
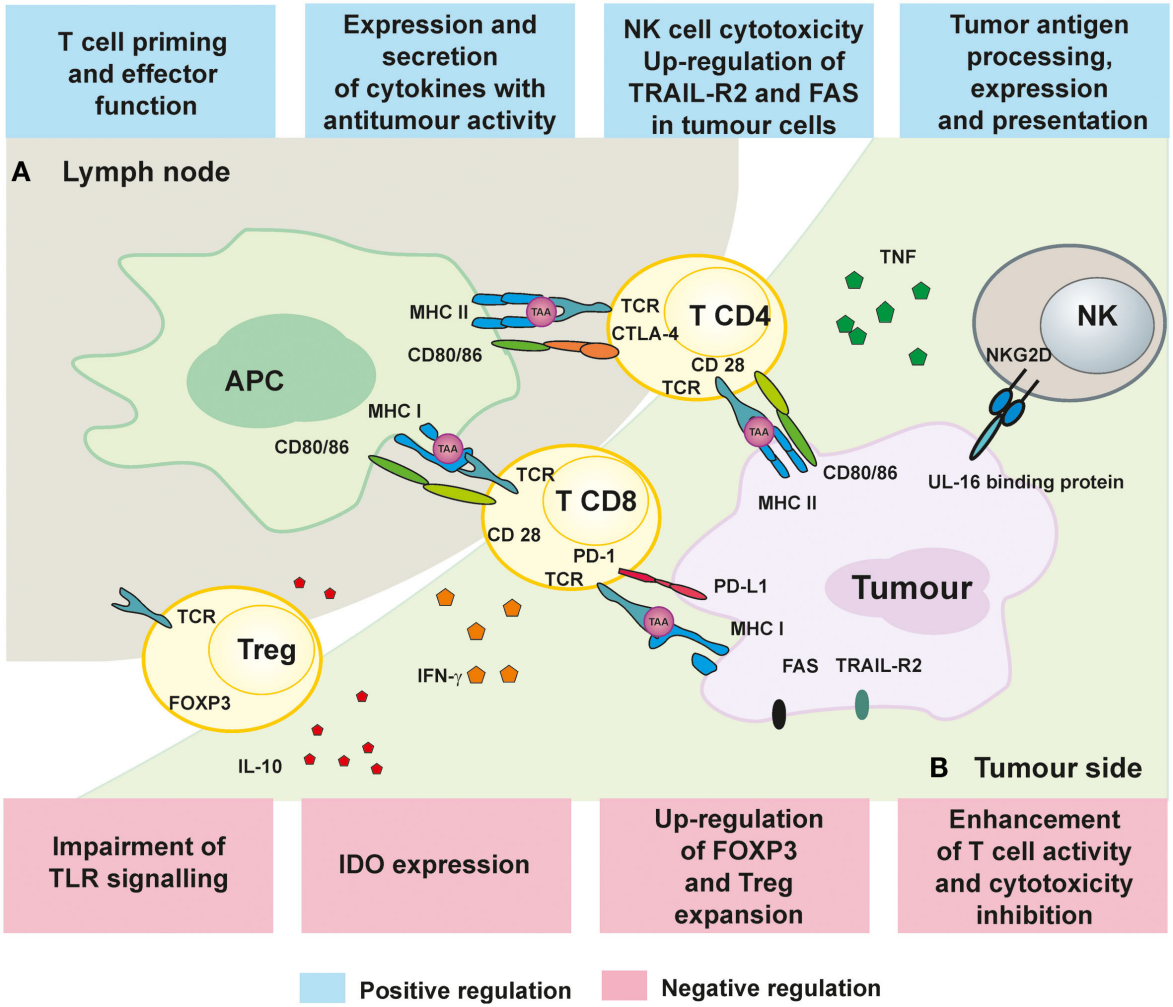
**Immunoregulatory mechanisms of epigenetic treatment**. **(A)** The influence of HDACi and hypomethylating drugs on APC presentation of TAA as well as activation and proliferation of T cell in lymph nodes. **(B)** Immune response in the tumors can be improved by epigenetic treatment by augmenting T and NK cell cytotoxicity and secretion of TNF and IFN-γ.

Epigenetic mechanisms include post-translational modifications of histone proteins affecting chromatin remodeling, DNA methylation, and regulation of gene expression by non-coding RNAs. A number of epigenetic events seem to play a pivotal role both in tumor progression and in avoiding immune recognition (67, 68). The most widely studied and best understood in terms of modulating immunity are DNA methylation and histone modifications (Figure 1).

Methylation occurs predominantly in CpG-rich regions called “CpG islands.” A characteristic feature of tumor cells is global hypomethylation of their genome, and hypermethylation of CpG island in promoter regions of various genes (69). Methylation of DNA involves covalent addition of methyl group to C5 of cytosine ring leading to generation of 5-methylcytosine (70). Methylation of promoter regions leads to recruitment of methyl-CpG-binding proteins that form chromatin-remodeling co-repressor complexes resulting in gene silencing (67). Methylation pattern in every cell is established and maintained by a family of proteins called DNA methyltransferases (DNMTs).

Histones acetylation is a reversible process of adding an acetyl moiety to lysine residues on histone proteins resulting in neutralization of their positive charge and impairing their interaction with negatively charged DNA. Therefore, acetylation increases the accessibility of regulatory proteins to DNA, enabling activation of various genes expression (71). This process may be reversed by the opposite activities of histones deacetylases (HDACs) that remove acetyl groups from histone proteins leading to recovery of N-terminal tail affinity to DNA strand. This results in chromatin condensation and suppression of transcription process.

### Influence of epigenetic therapy on tumor antigen expression

DNA methylation is one of the most important epigenetic ­mechanism regulating expression of genes responsible for recognition of tumor cells by host immune system. Particularly, this is relevant to methylation of gene promoters, which leads to silencing of TAA and APM proteins, enabling escape from tumor immune-surveillance (72).

The presence of TAA in cancer cells is a mandatory requirement for activation of effector CTLs. TAA can be divided into four different groups: (i) differentiation antigens, which are lineage-specific and expressed in tumor as well as in normal cells from which the tumors arise; (ii) overexpressed antigens, which are broadly expressed in many normal tissues, but present in tumor cells at higher levels; (iii) tumor-specific antigens, usually typical for individual tumors, resulting from genetic alterations; (iv) cancer–testis antigens (CTA) that are expressed in various types of malignant human tumors and are restricted in normal tissues to germ cells of the testis, with occasional expression in female reproductive organs (73). CTA are particularly susceptible to epigenetic regulation. They include melanoma-associated antigen (MAGE), NY-ESO-1, and SSX gene families as well as the GAGE/PAGE/XAGE superfamily. MAGE, GAGE, BAGE, SSX, and LAGE-1/NY-ESO-1 are frequently methylated and down-regulated in tumor cells. The CTA family also includes P1A antigen, one of the best known murine TAA, which is a homolog of human MAGE (74). P1A is an endogenous protein, initially identified in chemically induced mast cell-mastocytoma 815 (75). As a classical CTA, P1A is not expressed in normal tissues, but expressed only in placenta and testis. P1A epitopes are presented to T lymphocytes through MHC H2-k2d and may induce strong specific response of CTLs (76). Similar to human MAGE gene family, in several murine tumors P1A is not expressed as a result of methylation of the promoter regions (77).

### Immunoregulatory effects of drugs targeting epigenetic mechanisms

Drugs targeting epigenetic mechanisms can modulate expression of multiple genes including tumor suppressor genes, oncogenes, tumor associated antigens, as well as molecules involved in antigen presentation, co-stimulatory signaling and cytokines. Among different classes of genes described as epigenetically regulated, these encoding TAA are undoubtedly essential for T cell activation and tumor recognition by immune response. The expression of CTA can be restored by a number of hypomethylating agents (78, 79). Over 20 years ago, it was demonstrated that 5-aza-2′-deoxicitidyne (5-aza-dC) up-regulates MAGE-1 expression in tumor, but not in normal cells, and leads to HLA-A1-restricted lysis of tumor cells by CTLs (78). Further studies revealed that a variety of other CTA can be induced by either 5-aza-dC or other inhibitors of DNMTs (80–82). Methyltransferase inhibitors can also induce expression of MHC class I molecules. These effects result from the impact on both MHC genes as well as from regulation of virtually all components of the APM, including TAP1 and 2, proteasome subunits (81, 83). Moreover, antigen presentation can be augmented by up-regulation of type I and II interferons, their receptors, and components of the IFN-signaling pathways (84). Importantly, the effects of methyltransferase inhibitors are long-lasting as CTA are detectable for next several weeks after treatment and they are recognized by antigen-specific CD8^+^ T cells (77, 85, 86). Also histone deacetylase inhibitors (HDACi) may affect expression of TAA, but mostly by increasing or restoring expression level of proteins involved in antigen presentation. For example, trichostatin A (TCA) was shown to up-regulate or induce expression of TAP-1, TAP-2, tapasin, and LMP-2 in murine tumor cell lines (87, 88). Moreover, the TCA-mediated increase in MHC levels results in activation of adaptive immune response and inhibition of tumor growth in mice (88). Additionally, HDACi increase the expression of MHC class II and co-stimulatory molecules in human and mouse melanoma and trophoblast cell lines (89, 90). TCA, by means of activation of the pIII-CIITA promoter in neoplastic cells and induction of MHC class II expression, was also found to augment CD4^+^ T cells proliferation (91). However, the result of their action is complex, since they down-regulate one antigen, Muc1, and up-regulate another, NY-ESO-1, at the same time (92). Based on these findings, a great deal of interest has been generated in investigation of epigenetic therapy influence on antitumor immune response.

It was also demonstrated that HDACi can affect polarization of naive CD4^+^ T cells toward Th1 and Th2 subsets. Vorinostat by inhibiting STAT6 and TARC may impair the functions of CD4^+^ T cells, shifting the balance toward Th1 response (93). Also hypomethylating agents increase production of cytokines, including IL-2, TNF, and IFN-γ (94, 95). Epigenetic treatment facilitates killing of tumor cells by NK cells or CTLs through up-regulation of TRAIL-R2 and FAS in transformed cells (96, 97). Furthermore, enhancement of NK-mediated tumor cell death was also induced by TCA by up-regulation of UL-16-binding protein expression (a ligand for cytotoxicity NKG2D receptor) (98).

## Tumor Cell Recognition by Immune System after Photodynamic Therapy Enhanced by Epigenetic Treatment

The still elusive goal for effective cancer immunotherapy is to overcome tumor escape mechanisms and to trigger development of systemic adaptive antitumor immune response allowing for the control of distant metastases. As described above, PDT is an effective local treatment that induces acute local inflammatory response. However, development of systemic adaptive immune response after PDT strongly depends on the efficacy of presentation and recognition of tumor antigens by the immune cells (1). Various approaches have been examined to accelerate the priming phase of immune response after PDT. Induction of antitumor activity depends on activation of CD8^+^ T cells and administration of immature DCs into the PDT-treated tumors resulted in effective activation of T and NK cells (24). Also, the PDT effectiveness was improved by administration of adjuvants, such as glycated chitosan (99). Additionally, the role of expression level of MHC class I in PDT was evaluated in the treatment of patients with vulval intraepithelial neoplasia (VIN). In VIN lesions that respond to PDT, significant increase of CD8^+^ infiltration after treatment was observed when compared to non-responders. Interestingly, none of responding VIN patients showed any evidence of MHC class I down-regulation, whereas all of the cases of VIN lacking of MHC I failed to respond to PDT (100).

One of the approaches to increase immunogenicity of tumor cells focused on their genetic modification to enhance activation of CTLs by DCs. Introduction of foreign antigen, such as green fluorescence protein (GFP) to radiation-induced fibrosarcoma cells was observed to induce strong tumor-specific immune response allowing for long-term tumor control. Re-challenge experiments revealed that survived mice developed resistance to GFP-positive cells (101). These data are in line with another study demonstrating that the presence of β-galactosidase antigen in tumor cells is able to increase immunogenicity allowing PDT to elicit strong antitumor effects and long-term immunity to re-inoculated tumor cells (102). In this vein, the same authors have transfected tumor cells with a gene encoding P1A, a model CTA in the mouse. The presence of this antigen led to effective antitumor immune response that only developed when PDT was used and was found sufficient to prevent tumor growth when tumor cells were re-inoculated (74). Moreover, PDT was shown to enhance systemic immune responses to tumors in patients with Basal cell carcinoma. The immune recognition of cancer cell antigen – Hip 1 – was improved by PDT treatment (103).

**Figure 2:**
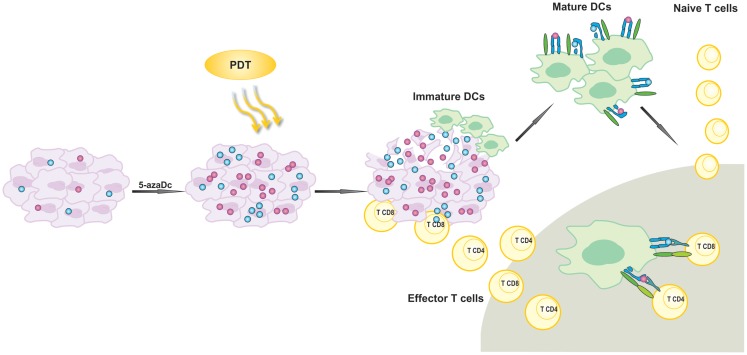
**Activation of antigen-specific antitumor immune response by photodynamic therapy (PDT)**. 5-aza-2′-deoxycitidine (5-aza-dC) up-regulates expression of silenced tumor-associated antigens (TAA). PDT leads to the release of TAA that are further phagocytosed by attracted to the tumor lesion of immature dendritic cells (DCs). Activated DCs migrate to local lymph nodes and present TAA-derived peptides in association with MHC molecules to T lymphocytes. T cells are activated and subsequently differentiate into effector cells homing to the tumor site in order to destroy residual tumor cells.

Considering that 5-aza-dC restores expression of CTA including P1A as well as MHC class I molecules (77), we sought to evaluate the antitumor effects of the combined treatment involving PDT and administration of 5-aza-dC. We have observed that treatment with 5-aza-dC alone restores expression of MHC class I molecules as well as induces expression of P1A antigen in four different murine tumor models and two strains of mice (104). Antitumor effects of 5-aza-dC were rather insignificant when used alone. However, when we combined 5-aza-dC with PDT, we observed prolonged complete antitumor responses in mice with EMT6 mammary tumors and CT26 colon adenocarcinomas and significant prolongation of survival in mice bearing 4T1 mammary tumors and LLC lung carcinomas. The antitumor effects of the combination treatment were strongly dependent on the presence of CD8^+^ CTLs as their depletion with monoclonal antibodies almost completely abrogated antitumor effects. On the other hand, CD4^+^ T cells played only a supportive role. We have also observed that the combined treatment led to expansion of IL-17-producing CD4^+^ T cells, which are known to stimulate CD8^+^. Intriguingly, pentamer staining for P1A-specific CD8^+^ T cell population revealed no significant changes in draining LNs and spleens between experimental groups. Moreover, all mice treated with PDT and 5-aza-dC that remained long-term tumor-free have rejected re-inoculated tumor cells even if the cells were P1A-negative. These findings suggest that the presence of P1A is not essential for the maintenance of long-lasting antitumor immunity. It is possible that 5-aza-dC combined with PDT may lead to increased expression and release of TAA in the PDT-treated microenvironment. Together with PDT-induced inflammation and the release of DAMPs, the combination treatment would confer better antigen presentation of P1A. Improved tumor recognition by immune cells can further expand the repertoire of antigen-specific T cells thereby increasing the immunogenic outcome of PDT. Thus, it can be hypothesized that PDT of tumors with TAAs up-regulated by 5-aza-dC can induce concomitant immunity to other subdominant and possibly weakly immunogenic antigens and facilitate epitope spreading. Immunity to the latter can sustain antitumor activity in mice. To summarize, our findings demonstrate that treatment combining 5-aza-dC with PDT leads to local cytotoxic effects accompanied by the release of induced TAAs allowing for development of antitumor immune response and long-term survival (Figure 2). Based on these findings, we hypothesize that inhibition of DNA methylation could unleash stronger immune response against TAA in tumor-bearing mice undergoing PDT.

## Author Contributions

MW, AM, and JG reviewed relevant literature. MW, AM, and JG drafted the manuscript. JG revised the manuscript and supervised MW and AM. MW and AM designed the figure. All authors read and approved the final manuscript.

## Conflict of Interest Statement

The authors declare that the research was conducted in the absence of any commercial or financial relationships that could be construed as a potential conflict of interest.
